# Coverage, Timing, Safety, and Determinants of Neonatal Intensive Care Unit Admission in Newborns Receiving Nirsevimab Prophylaxis for Respiratory Syncytial Virus

**DOI:** 10.3390/pathogens15080813

**Published:** 2026-08-01

**Authors:** Maria Costantino, Valentina Giudice, Anna Maria Della Corte, Silvia Pecoraro, Anna Rita Frascogna, Giuseppe Marchesano, Sabino Moschella, Carmela Alfano, Federica Santaniello, Carmine Brengola, Giuseppina Napoletano, Virginia Caputo, Antonio Luciano, Assunta Santullo, Luca Pierri, Federica Aiello, Maria Carmen De Caro, Luciana Catena, Concetta Sarnataro, Francesco De Caro, Maria Grazia Corbo

**Affiliations:** 1 Department of Medicine, Surgery, and Dentistry, University of Salerno, 84081 Baronissi, Italyfdecaro@unisa.it (F.D.C.); 2 University Hospital “San Giovanni di Dio e Ruggi d’Aragona”, 84131 Salerno, Italy; 3 Faculty of Medicine, Sofia University “St. Kliment Ohridski”, 1407 Sofia, Bulgaria; 4 Neonatal Intensive Care and Neonatology Unit (U.O.S.D.), Department of Emergency and Acceptance (DEA), Umberto I Hospital, Nocera Inferiore–Pagani–Scafati, Salerno Local Health Authority (ASL Salerno), 84014 Nocera Inferiore, Italy; g.marchesano@aslsalerno.it (G.M.); v.caputo17@studenti.unisa.it (V.C.); 5 Department of Neonatology and Neonatal Intensive Care Unit, San Giuseppe Moscati Hospital, 83100 Avellino, Italy; 6 Department of Pediatrics and Neonatology (UOC), Martiri Villa Malta Hospital, Salerno Local Health Authority (ASL Salerno), 84087 Sarno, Italy; 7 Neonatal Intensive Care and Neonatology Unit, AORN—IRCCS Santobono-Pausilipon Children’s Hospital, 80129 Naples, Italy

**Keywords:** RSV infection, AEFIs, nirsevimab, NICU admission, prematurity, prophylaxis coverage

## Abstract

**Background:** Nirsevimab, a long-acting monoclonal antibody targeting respiratory syncytial virus (RSV), has recently been introduced as universal prophylaxis in newborns and infants. Although clinical trials have shown high efficacy and a favorable safety profile, real-world evidence remains limited. **Objectives:** Our Italian multicenter observational study included newborns eligible for nirsevimab prophylaxis during the 2025–2026 season and aimed to assess nirsevimab coverage, timing of administration, and short-term safety in routine clinical practice. The association between perinatal and environmental factors and neonatal intensive care unit (NICU) admission was also investigated, while RSV occurrence was evaluated as a descriptive and exploratory outcome. **Methods:** Eligible newborns were from four Italian hospitals during the 2025–2026 RSV season. We assessed nirsevimab coverage, timing of administration, RSV incidence, adverse events, and selected neonatal, perinatal, and maternal risk factors. **Results:** Among 1554 eligible newborns, 1462 received nirsevimab, corresponding to a coverage rate of 94.0%. The median age at administration was 3.0 days (IQR 2-6 days), with significantly delayed prophylaxis in preterm compared with term infants. During the observation period, two laboratory-confirmed RSV-associated bronchiolitis cases occurred, corresponding to a cumulative incidence of 0.13%. One case occurred in the immunized group and one in the non-immunized group. No adverse events following immunization were recorded. **Conclusions:** Nirsevimab prophylaxis achieved high coverage and showed a reassuring short-term safety profile in this real-world multicenter cohort. RSV infections were uncommon, and their very limited number prevents definitive conclusions regarding comparative effectiveness between immunized and non-immunized newborns. Prematurity was associated with delayed administration and greater need for intensive care, underscoring the importance of timely prophylaxis in vulnerable newborns.

## 1. Introduction

Respiratory syncytial virus (RSV) is one of the leading causes of lower respiratory tract infections during the first year of life and is responsible for a substantial healthcare burden on pediatric services, with high rates of emergency care access and hospitalizations. Bronchiolitis is the most common clinical manifestation of RSV infection and represents one of the most frequent causes of hospitalization among infants. In Italy, recent epidemiological estimates indicate that a single RSV epidemic season during the first year of life is associated with more than 200,000 medically attended lower respiratory tract infections, over 13,000 hospitalizations, more than 2000 intensive care unit admissions, and approximately 16 deaths, resulting in a considerable increase in the national healthcare economic burden [1]. RSV affects infants born during the epidemic season as well as those born throughout the year, highlighting the need for preventive strategies capable of providing protection during the first months of life. Although clinical trials have demonstrated that nirsevimab, a long-acting monoclonal antibody targeting the RSV fusion protein, has high efficacy and a favorable safety profile in preventing medically attended RSV infections [2,3,4,5,6,7], evidence from real-world clinical practice remains limited [8,9,10]. Real-world data are essential for assessing the impact of large-scale immunization strategies in routine clinical practice and across heterogeneous populations with diverse perinatal and environmental characteristics. 

Currently, prophylaxis against RSV infection in neonates and infants includes the use of monoclonal antibodies as an effective strategy for direct protection during early life. Palivizumab, characterized by a short half-life and the need for monthly intramuscular administrations (up to five doses during the RSV season), was primarily reserved for high-risk infants, such as preterm newborns and those with specific underlying medical conditions; however, it has been discontinued since December 2025 and is no longer used in clinical settings [11,12,13]. More recently, the introduction of long-acting monoclonal antibodies has substantially changed the landscape of RSV prevention. Nirsevimab provides protection for at least five months following a single intramuscular injection and is indicated for the prevention of RSV lower respiratory tract disease during the first RSV season in both term and preterm infants [14]. In addition, clesrovimab, another long-acting monoclonal antibody administered as a single intramuscular dose, has recently been approved for the prevention of RSV lower respiratory tract disease in neonates and infants during their first RSV season, further expanding the available preventive options [15,16]. Although randomized clinical trials provide robust evidence on efficacy and safety, real-world studies are essential to evaluate the impact of preventive strategies under routine healthcare conditions and across heterogeneous populations. This is particularly relevant for recently introduced preventive interventions, where post-authorization observational studies can capture outcomes in broader and more diverse populations that are often underrepresented in clinical trials [8,9,10,17,18].

Among environmental determinants, maternal tobacco smoke exposure during pregnancy has been consistently associated with adverse respiratory and perinatal outcomes, including impaired fetal growth, an increased risk of preterm birth and low birth weight, as well as potential deleterious effects on neonatal immune and respiratory development. Both active maternal smoking and exposure to secondhand tobacco smoke have also been associated with an increased risk of respiratory infections during the first year of life [19,20,21]. Moreover, the mode of delivery has been associated with differences in the initial microbial colonization of newborns, with cesarean delivery being linked to reduced microbiota diversity and potential alterations of the gut–lung axis compared with vaginal delivery, potentially affecting immune system development [22,23,24,25]. Furthermore, maternal parity may serve as an indirect indicator of household exposure to respiratory pathogens, particularly through the presence of older siblings, who represent one of the main sources of household RSV transmission [26,27]. For this reason, post-marketing evaluation of nirsevimab in real-world clinical practice is essential to better understand its efficacy and safety under routine use conditions.

In this multicenter observational study, we aimed to assess nirsevimab coverage, timing of administration, and short-term safety in a cohort of eligible newborns managed in routine clinical practice. In addition, we investigated the association between selected early-life risk factors, including maternal smoking during pregnancy, mode of delivery, and maternal parity, and the likelihood of neonatal intensive care unit (NICU) admission following nirsevimab administration. Finally, we provide a descriptive overview of RSV incidence in this real-world setting.

## 2. Materials and Methods

### 2.1. Population and Study Design

This multicenter observational study was conducted across four Italian hospitals in the Campania Region: C1, University Hospital “San Giovanni di Dio e Ruggi d’Aragona”, Salerno; C2, “Umberto I” Hospital, Nocera Inferiore-Pagani-Scafati, Salerno; C3, “Martiri Villa Malta” Hospital, Sarno, Salerno; and C4, Neonatal Intensive Care Unit (NICU), “San Giuseppe Moscati” Hospital, Avellino. The study was conducted during the 2025–2026 RSV prophylaxis campaign (from October 2025 to April 2026) and included a cohort of 1554 neonates eligible for passive immunization against RSV with nirsevimab according to standard clinical practice across the four participating centers (C1, N = 560; C2, N = 611; C3, N = 270; and C4, N = 113). According to nirsevimab administration status, neonates were divided into an immunized cohort that received nirsevimab at the participating centers (N = 1462) and a non-immunized group (N = 92) whose parents declined nirsevimab administration. 

Inclusion criteria were: neonates born or admitted to the participating centers during the study period and assessed for nirsevimab prophylaxis according to local and national clinical practice; availability of complete clinical documentation; and written informed consent from parents or legal guardians. Exclusion criteria were: neonates with missing or incomplete medical records; those who received nirsevimab outside the participating centers; and lack of parental or legal guardian consent. The study was conducted in accordance with the principles of the latest revision of the Declaration of Helsinki and with the current WHO guidelines for surveillance of adverse events following immunization (AEFI) [28] and was approved by the local Ethics Committee “Campania 2”, Naples, Italy (approval no. 0027864, 22 May 2026). Written informed consent for nirsevimab administration was obtained from parents or legal guardians as part of standard clinical practice. Anonymized data were retrospectively extracted from medical records using a structured case report form (CRF), ensuring that no personally identifiable information was collected. The description of study variables, outcomes, and measurement methods was consistent with the Strengthening the Reporting of Observational Studies in Epidemiology (STROBE) guidelines for observational studies [29].

### 2.2. Data Collection 

Collected data included demographic and perinatal variables: gestational age (GA, weeks), birth weight (grams), sex, mode of delivery (vaginal or cesarean section), comorbidities, maternal smoking (active and passive exposure) during pregnancy, and maternal parity (categorized as primiparous or multiparous). For multiparous women, the number of previous children (number of older siblings) was also recorded as a continuous variable and used as an additional indicator of household exposure to respiratory pathogens, with primiparous women coded as 0 by definition. In addition, dates of birth and nirsevimab administration were collected. GA and birth weight were classified according to standard international neonatal criteria: preterm, with GA < 37 weeks; term, with GA ranging from 37 to 42 weeks; and post-term, with GA ≥ 42 weeks. Birth weight was categorized as low birth weight (LBW) < 2500 g, normal birth weight (NBW) ranging from 2500 to 4000 g, and high birth weight (HBW) > 4000 g.

Occurrence of AEFI and serious AEFI (SAEFI) was recorded, together with type, severity, duration, and time of onset after nirsevimab administration. Events were classified in accordance with Italian and European pharmacovigilance guidelines [30,31,32]. RSV case ascertainment was performed according to a predefined institutional protocol. All neonates presenting to the emergency department or requiring hospital admission with respiratory symptoms underwent systematic virological testing. RSV infection was confirmed using RT-PCR at C4, the FilmArray Respiratory Panel (multiplex molecular assay) or a rapid antigen test at C2, a molecular test at C1, and a rapid RSV antigen test at C3. Only laboratory-confirmed RSV-positive cases were included in this analysis. Testing was performed regardless of nirsevimab immunization status across all participating centers. For the assessment of RSV occurrence over time, individual follow-up data were also collected, including date of birth, date of laboratory-confirmed RSV infection for cases, and date of last follow-up. These variables were used to define the observation period for each infant and to calculate individual follow-up duration and total person–time at risk.

### 2.3. Endpoints

The primary endpoint was the occurrence of RSV infection during the observation period, described as cumulative incidence and as incidence density. In neonates receiving nirsevimab prophylaxis, episodes occurring at least 14 days after administration were classified as breakthrough post-immunization infections, while infections occurring within 14 days of administration were not classified as breakthrough infections, as they may have been acquired during the incubation period before the development of protective nirsevimab concentrations. The secondary endpoint included the assessment of the short-term safety of nirsevimab prophylaxis. Safety surveillance was passive. Parents or caregivers were instructed to report any adverse events occurring within 30 days after nirsevimab administration to the vaccination service or to their primary care pediatrician. No scheduled follow-up visits, telephone interviews, or other active surveillance procedures were conducted.

Outcomes related to the implementation of the prophylaxis strategy were also evaluated, including adherence to nirsevimab prophylaxis, defined as the proportion of immunized neonates among all eligible neonates across participating centers; prophylaxis hesitancy, defined as parental refusal of nirsevimab administration after counseling regarding its purpose, expected effectiveness, and safety profile; and timeliness of prophylaxis, calculated as the interval between date of birth and date of immunization, and used to describe the temporal distribution of administration. 

An exploratory analysis was also conducted to investigate neonatal, perinatal, and early maternal risk factors and included maternal smoking during pregnancy (active smoking and/or exposure to passive smoking, collected through maternal history), mode of delivery (vaginal delivery, operative vaginal delivery, or cesarean section, dichotomized as cesarean section versus all other modes of delivery in the regression analysis given the small number of operative vaginal deliveries), and maternal parity.

### 2.4. Statistical Analysis

No formal a priori sample size calculation was performed, as this was an exploratory, observational study evaluating the real-world implementation of nirsevimab prophylaxis rather than a confirmatory efficacy trial. For this reason, the sample size corresponds to the entire population of neonates eligible for nirsevimab prophylaxis at the four participating centers during the 2025–2026 RSV season. Statistical analyses were performed using SPSS 23.0. Categorical variables were presented as absolute frequencies and percentages, while continuous variables were described as mean ± standard deviation (SD). For variables with missing data, percentages were calculated using the number of non-missing observations, and the number of missing values was explicitly reported. The incidence of RSV infection was calculated as the proportion of infants with confirmed infection among the total population, according to the specific reference cohort (immunized and/or eligible). Because enrollment occurred over a staggered time window during the 2025–2026 RSV prophylaxis campaign, follow-up time was calculated individually for each infant. Observation time started at birth and ended at the earliest of laboratory-confirmed RSV infection or administrative censoring on 30 April 2026. For infants without RSV infection, follow-up was calculated as the interval between date of birth and 30 April 2026; for infants with RSV infection, follow-up was calculated as the interval between date of birth and date of RSV diagnosis. Follow-up duration was summarized as the median, interquartile range (IQR), and range. Total person–time at risk was obtained by summing individual follow-up times across the cohort and was expressed in person-years. RSV occurrence was also reported as incidence density, calculated as the number of RSV events divided by the total accrued person–time, with 95% confidence intervals (95% CI). Clinical efficacy was expressed as risk ratio (RR) and relative risk reduction (RRR), with 95% confidence intervals (95%CI). Differences between groups for continuous variables were analyzed using independent-samples t-tests or Mann–Whitney U tests, depending on data distribution. Safety was assessed using AEFI and SAEFI frequency, reported as proportions with corresponding 95%CI. A cumulative frequency analysis of AEFI within 30 days after nirsevimab administration was also performed. Uptake of prophylaxis and hesitancy were analyzed as proportions of the overall cohort and compared across participating centers using tests for proportions. Timeliness of nirsevimab administration was described as a continuous variable (days of life) and compared between subgroups (preterm vs. term infants) using the Mann–Whitney U test. To account for potential confounders of administration timing, a multivariable linear regression model was performed, with age at administration as the dependent variable and preterm status, participating center, birth season, and NICU admission as independent predictors. An exploratory analysis of the association between risk factors (maternal smoking exposure, mode of delivery, and maternal parity) and clinical and safety outcomes was conducted using univariate logistic regression. In a sensitivity analysis, the number of previous children was also included as an additional covariate in the multivariable model for NICU admission to assess whether household exposure was independently associated with outcomes. Results were reported as odds ratios (OR) with 95% CI. A p < 0.05 was considered statistically significant.

## 3. Results

### 3.1. Study Population

Of the 1554 neonates eligible for nirsevimab prophylaxis, 1462 (94%) received immunization across the participating centers, while 92 (6%) were not immunized due to parental refusal. The baseline characteristics of the study population, stratified by immunization status, are summarized in Table 1. Overall, immunized and non-immunized neonates had comparable baseline characteristics, with no statistically significant differences in sex distribution, GA, birth weight, mode of delivery, or maternal parity (all p > 0.05). Among the 1341 immunized neonates with available data on the number of previous children, the median number was 0 (IQR 0–1; range 0–7), reflecting a large proportion of primiparous women. Among the 672 multiparous women with available data, the median number of previous children was 1 (IQR, 1–2). In the non-immunized group, among the 38 subjects with available data, the median number of previous children was 1 (IQR 0–2; range 0–4). The immunized cohort consisted of 53% males (N = 771) and 47% females (N = 691), with a mean GA of 38 ± 2.0 weeks (range, 26–42) and a mean birth weight of 3130 ± 560 g. Preterm neonates (N = 163, 11.2%) had a mean GA of 34 ± 2.1 weeks (range, 26–36) and a mean birth weight of 2221 ± 623 g, whereas term neonates (N = 1296, 88.6%) had a mean GA of 39 ± 1.1 weeks (range, 37–41) and a mean birth weight of 3243 ± 433 g. Three post-term neonates (0.2%) were identified and excluded from inferential analyses due to their very small number.

No AEFIs or serious AEFIs (SAEFIs) were reported among the 1462 nirsevimab recipients during the 2025–2026 season, including both term and preterm neonates (Table 1), with a frequency of 0% (exact 95% CI, 0.00–0.21%).

### 3.2. Season-Wide RSV Infection Rate 2025–2026

During the 2025–2026 epidemic season, two cases of laboratory-confirmed RSV bronchiolitis were identified in the entire cohort, corresponding to an overall incidence of 0.13% (2/1554; 95% CI, 0.04–0.47). The median follow-up was 115 days (IQR, 77–164 days; range, 30–473 days), corresponding to a total of 512.2 person-years of observation, and overall RSV incidence density was 3.91 per 1000 person-years (95% CI, 0.47–14.10). The distribution across participating centers was heterogeneous: one case was recorded at center C1 (0.18%, 1/560) and one case at center C3 (0.37%, 1/270), whereas no RSV infections were observed at centers C2 and C4. One case occurred in the immunized group (0.07%; 95% CI, 0.01–0.39), representing a breakthrough infection, and one case in the non-immunized group (1.09%; 95% CI, 0.19–5.90); thus, differences were not statistically significant (Fisher’s exact test, p = 0.115). The estimated RR was 0.063 (95% CI, 0.004–0.998); however, given the occurrence of only one RSV event in each group, this estimate is highly imprecise. The corresponding RRR was 93.7% (95% CI, 0.2–99.6%), the absolute risk reduction was 1.02% (95% CI, −1.09% to 1.09%), and the number needed to immunize was 98 (95% CI, 92–98). These estimates should be considered exploratory and hypothesis-generating only and not interpreted as definitive measures of vaccine efficacy.

The immunized 50-day-old neonate who tested positive for RSV had several clinical characteristics potentially associated with increased vulnerability, including prematurity (GA, 33 weeks), twin pregnancy, viral coinfection with human coronavirus 229E, and previous admission to the NICU. The non-immunized 98-day-old RSV-positive neonate was born at term, had no evidence of viral coinfections, and had been discharged from the well-baby nursery. Both neonates were male and required respiratory support with high-flow nasal cannula (HFNC). The immunized neonate required HFNC for one day with a flow of 2 L/kg and an FiO_2_ of 0.21, without corticosteroid therapy, and had a maximum Silverman score of 4. The non-immunized neonate required HFNC for six days with a flow of 7 L/kg and an FiO_2_ of 0.30, received oral corticosteroid treatment for seven days, and had a maximum Silverman score of 5. The immunized neonate was discharged on day 9, whereas the non-immunized neonate was discharged on day 7. These clinical observations and incidence estimates should be interpreted with caution, as they are based on an extremely limited number of RSV infections.

### 3.3. Outcomes Related to Implementation of Prophylaxis and Exploratory Analyses

The median age at nirsevimab administration in the entire cohort was 3.0 days (IQR, 2–6 days; mean 6.0 ± 16 days). Among preterm neonates, the median age at administration was 9.0 days (IQR, 4–25 days; mean 20.6 ± 36 days), while in term neonates it was 3.0 days (IQR, 2–5 days; mean 4.2 ± 10 days). Prophylaxis was therefore significantly delayed in preterm infants compared with term neonates (Mann–Whitney U test, p < 0.001). Regarding the timeliness of prophylaxis, in neonates born vaginally, a significant difference was observed between preterm and term infants (median 6.5 days, IQR 4–15 days, vs. median 3.0 days, IQR 2–4 days; Mann–Whitney U test, p = 0.002). In the cesarean delivery group, nirsevimab administration was likewise significantly delayed in preterm compared with term neonates (median 10.0 days, IQR 4–30 days, vs. median 3.0 days, IQR 2–6 days; Mann–Whitney U test, p < 0.001). 

Because the timing of administration may also be influenced by center-specific organizational practices, birth season, and NICU hospitalization, an adjusted linear regression model was performed with age at nirsevimab administration (days) as the dependent variable and preterm status, participating center, birth season, and NICU admission as predictors. After adjustment, preterm status remained significantly associated with delayed administration (adjusted beta coefficient, +8.4 days; 95% CI, 6.1–10.7; p < 0.001), indicating that the delay in prophylaxis among preterm neonates was not linked to center guidelines, birth season, or NICU hospitalization.

Among the immunized neonates with available data on maternal smoking exposure, 395 mothers (27.0%) reported any exposure to tobacco smoke during pregnancy, whereas 1067 (73.0%) had no exposure. The prevalence of smoking exposure was similar between vaginal delivery and cesarean section (28.2%, 220/781 vs. 25.7%, 175/681; p = 0.32). Among neonates born to mothers exposed to smoking, the distribution of discharge destination was similar between vaginal and cesarean delivery. In contrast, among neonates born to non-exposed mothers, discharge to the well-baby nursery was more frequent after vaginal delivery, whereas admission to the NICU was more common in the cesarean section group.

**Figure 1:**
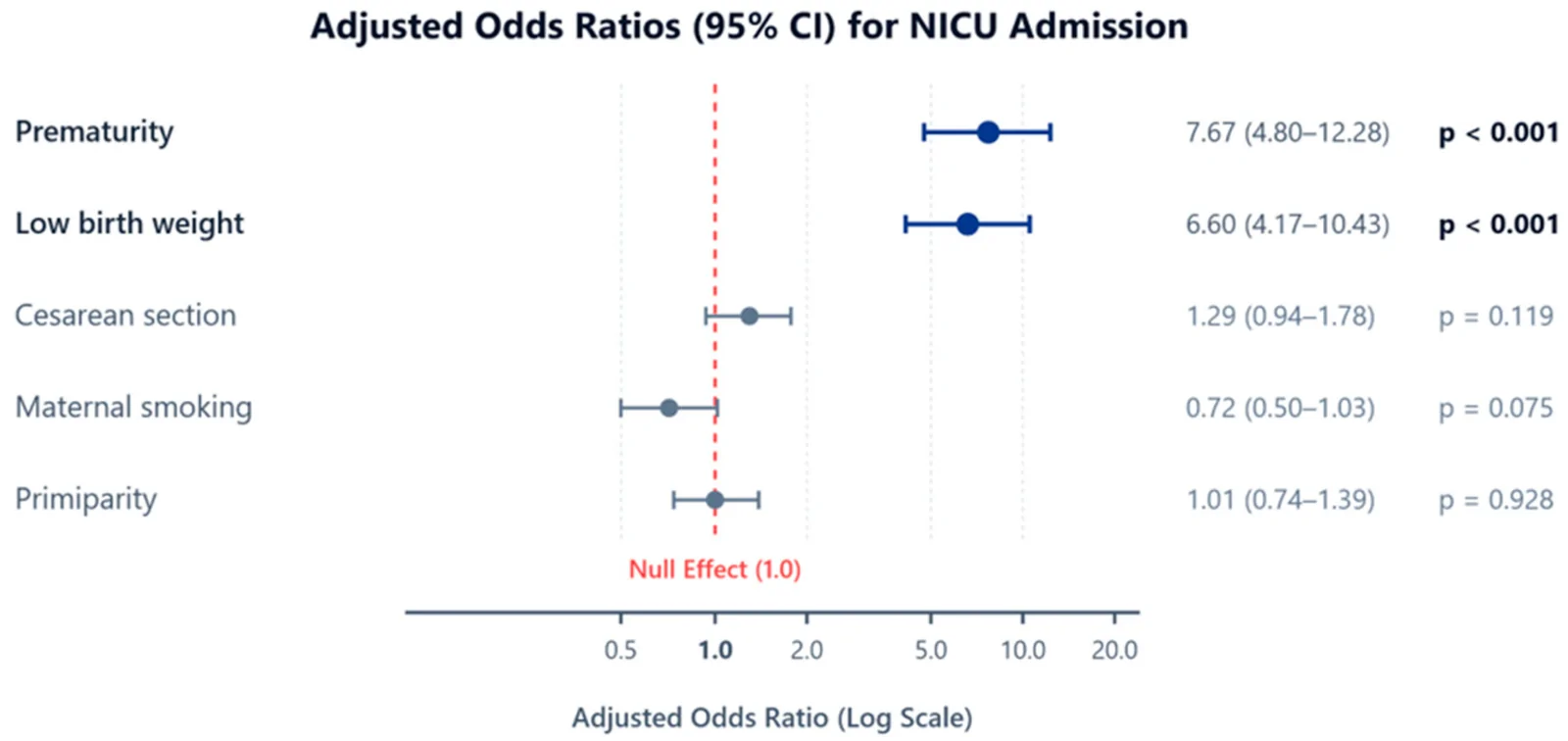
Forest plot of adjusted odds ratios for the association between perinatal/maternal factors and NICU admission. Abbreviations. OR, odds ratio; CI, confidence interval.

Overall, neonates admitted to the NICU had significantly lower GA and birth weight compared with those not admitted to the NICU, with a markedly higher prevalence of prematurity in the NICU group. In multivariable analysis, the main determinants of NICU admission were prematurity and low birth weight; maternal smoking exposure was not independently associated with the outcome, whereas cesarean delivery was associated with a modest and non-significant increase in NICU admission risk. Because prematurity (GA < 37 weeks) and low birth weight (<2500 g) were strongly correlated, collinearity diagnostics were inspected, and the variance inflation factors were within acceptable limits (<5). To further limit collinearity, the model using continuous GA and birth weight was adopted as the primary specification. In an alternative model using GA and birth weight as continuous variables, GA remained a strong independent predictor (mean ± SD, 36.3 ± 3.0 weeks vs. 38.8 ± 1.2 weeks, NICU admitted vs. non-admitted; p < 0.001), as well as birth weight (mean ± SD, 2665 ± 777 g vs. 3242 ± 425 g, NICU admitted vs. non-admitted; p < 0.001). These findings were also confirmed by multivariable logistic regression analysis (Figure 1). In this model adjusted for prematurity, low birth weight, cesarean section, maternal smoking, and primiparity, GA remained a strong independent predictor (adjusted OR, 0.60 per additional week; 95% CI, 0.53–0.68; p < 0.001), as did birth weight (adjusted OR, 0.999 per gram; p < 0.001), while cesarean section and smoking were not associated. 

In a sensitivity analysis, the number of previous children was added as an additional covariate to the multivariable model for NICU admission. The number of previous children was not independently associated with NICU admission, both in the dichotomous model (adjusted OR, 1.06 per additional child; 95% CI 0.90–1.26; p = 0.47) and in the continuous GA/birth weight model (adjusted OR, 0.98 per additional child; 95% CI 0.83–1.16; p = 0.85). Estimates for prematurity, low birth weight, cesarean section, and maternal smoking remained substantially unchanged, indicating that the household exposure indicator did not confound our observed associations.

## 4. Discussion

The present study evaluated the implementation of nirsevimab prophylaxis in a multicenter cohort of eligible neonates, describing both the overall uptake of the preventive strategy and selected clinical and perinatal factors associated with timing of administration and discharge destination. The results show high prophylaxis coverage (94%), with a low rate of non-uptake mainly due to parental refusal after informed counseling [33,34,35]. This finding suggests a good acceptability of prophylaxis in the studied population and confirms the crucial role of information provided to families in the decision-making process.

A relevant finding from the analysis concerns the timing of nirsevimab administration. In preterm neonates, immunization was significantly delayed compared with term infants, both after vaginal delivery and cesarean delivery. This finding can plausibly be explained by the greater clinical complexity of preterm neonates, who more frequently require prolonged hospital care and, in the case of cesarean delivery, may experience a more complex postnatal course [36,37]. The observed difference highlights how prematurity continues to represent a factor associated with less timely implementation of preventive strategies.

The analysis of the clinical characteristics of immunized neonates also showed that prematurity is strongly associated with cesarean delivery and with significantly lower birth weight, with a higher frequency of low birth weight among preterm infants. These findings are consistent with the underlying pathophysiology and with the well-established association between preterm birth, reduced neonatal maturity, and increased need for hospital care [38]. In this context, the assessment of discharge destination is particularly relevant, as admission to the NICU was strongly influenced by prematurity and low birth weight, whereas cesarean delivery and maternal smoking did not appear to be independent factors once other covariates were accounted for.

Regarding maternal smoking exposure, the observed prevalence was relatively high but similar between vaginal delivery and cesarean delivery. Multivariable analysis did not show an independent association between maternal smoking and NICU admission, suggesting that its effect may be partly mediated by other clinical factors, particularly prematurity and low birth weight [39]. This finding is noteworthy, as it indicates that smoking, although an important obstetric and perinatal risk factor, does not emerge as an independent predictor of the outcome in the final model. The apparent protective direction of the association between maternal smoking and NICU admission (unadjusted OR < 1) is biologically implausible and is most plausibly explained by residual confounding and selection effects within this cohort; in particular, mothers who smoked were not randomly distributed across gestational age and mode of delivery, and smoking was more frequent among women with term vaginal deliveries, who are at lower baseline risk of NICU admission. Conditioning on these correlated variables in the multivariable model may therefore have introduced collider stratification or selection bias, distorting the smoking estimate. For these reasons, the smoking coefficient should not be interpreted as a true protective effect of smoking on NICU admission.

From a safety perspective, no AEFIs were reported among infants who received nirsevimab prophylaxis, supporting its favorable tolerability profile. Because safety surveillance relied on passive reporting, mild or transient AEFIs may have been under-ascertained. Therefore, the absence of reported adverse events should be interpreted as a reassuring finding rather than definitive evidence of the absence of events, particularly those that are rare or delayed, which require larger populations and longer follow-up periods [33,40,41].

Regarding RSV infection outcomes, two laboratory-confirmed RSV bronchiolitis cases were identified during the surveillance season, corresponding to a low cumulative incidence of medically attended RSV infection, a total of 512.2 person-years of observation, and an overall RSV incidence density of 3.91 per 1000 person-years (95% CI, 0.47–14.10). One case occurred in the immunized group and one in the non-immunized population. Although the estimated RR suggests a lower occurrence of RSV infection among immunized neonates, this estimate was based on only two events and remains highly imprecise. Moreover, in the absence of a historical comparator, the observed RSV incidence cannot be directly attributed to nirsevimab prophylaxis, as seasonal epidemiological variation and other factors may have contributed to the observed findings. Therefore, our comparative analysis should be considered exploratory and hypothesis-generating rather than as evidence of a definitive preventive effect of nirsevimab. Clinical characteristics of the two RSV-positive neonates provide additional descriptive information. The breakthrough infection occurred in a preterm neonate with clinical vulnerability factors, including twin pregnancy, previous NICU admission, and viral coinfection. Conversely, the non-immunized RSV-positive neonate was born at term and had no documented coinfections. Both infants required respiratory support, although these observations are based on only two cases and do not allow conclusions regarding differences in disease severity or the potential impact of immunization on the clinical course. Our results support the feasibility and organizational implementation of the RSV prophylaxis strategy, with high coverage and successful integration into routine neonatal care. However, the very low number of RSV events prevents the definitive estimation of nirsevimab efficacy. Although the person–time strengthens the interpretation of RSV occurrence in the presence of staggered enrollment, it does not overcome the limited precision related to the very small number of observed events. 

The study has several limitations that should be considered when interpreting the results. First, this is an observational analysis, which does not allow for the establishment of a causal relationship between prophylaxis and reduction in RSV infection, nor between the evaluated risk factors and clinical outcomes. The selection of variables included in the multivariable model was guided by clinical plausibility, but no formal directed acyclic graph (DAG)-based causal framework was used to identify confounders; unmeasured confounders and selection bias may therefore still influence the observed associations. For instance, the apparently protective univariate association between maternal smoking and RSV-related outcomes (OR = 0.63) may reflect unmeasured confounding or selection bias rather than a true effect. In addition, the multicenter design, while representing strength in terms of generalizability, may have introduced heterogeneity in organizational practices, clinical management, and timing of prophylaxis administration across participating centers. Moreover, although RSV testing was systematically performed in neonates presenting with respiratory symptoms at emergency department visits or hospital admission, infections with mild clinical presentations managed at home without medical assessment were not captured. Therefore, the reported incidence reflects medically attended, laboratory-confirmed RSV infections rather than the real burden in the general neonatal population. Another limitation is the extremely small number of RSV infection cases observed during the surveillance season. This reduced the statistical power of comparative analyses and resulted in wide uncertainty around effectiveness estimates. For this reason, reported risk estimates should be interpreted as exploratory and hypothesis-generating only. In addition, because follow-up duration differed across infants, cumulative incidence alone may provide an incomplete representation of RSV occurrence; thus, incidence density was also reported. Similarly, the absence of adverse events does not allow the exclusion of rare or delayed events, which may only emerge in larger cohorts or with longer follow-up. Regarding maternal smoking, it should be noted that exposure was collected through maternal history and is therefore potentially subject to information bias or underreporting. Moreover, the variable included both active and passive smoking, making the measure broader but less specific with respect to individual exposure components. Finally, the exploratory risk factor analysis was based on selected variables and cannot exclude the potential role of other confounders that were not available or not included in the dataset.

## 5. Conclusions

In conclusion, our study demonstrates high uptake of nirsevimab prophylaxis among eligible neonates, with good acceptability among families and a reassuring short-term safety profile in this real-world multicenter cohort. Prematurity and low birth weight were strongly associated with the need for neonatal intensive care and with less timely administration of prophylaxis. Maternal smoking exposure, although common, was not an independent predictor of NICU admission. Overall, our findings support the feasibility and successful implementation of the nirsevimab prevention strategy in routine neonatal care and highlight the importance of ensuring timely administration, particularly in preterm and other high-risk neonates. The low number of RSV events observed during the surveillance period does not allow for definitive conclusions regarding prophylactic efficacy, and the observed RSV incidence should be interpreted cautiously. Future studies with larger sample sizes, longer follow-up, and appropriate comparator groups are needed to more robustly assess the impact of prophylaxis on RSV-related clinical outcomes.

## Figures and Tables

**Table 1:** Baseline characteristics of the study population according to immunization status.

Characteristics	Immunized N = 1462	Non-Immunized N = 92	p Value
M/F, n (%)	771 (53)/691 (47)	47 (51)/45 (49)	0.842
Birth weight, g (mean ± SD)	3130 ± 560	3086 ± 500	0.417
GA, weeks, mean ± SD (range)	38 ± 2.0 (26–42)	38 ± 1.9 (29–41)	0.413
Preterm, n (%)	163 (11.2)	13 (14.1)	0.485
Term, n (%)	1296 (88.6)	79 (85.9)	0.485
Post-term, n (%)	3 (0.2)	0 (0)	-
Birth weight category, n (%)			0.299 *
LBW	166 (11.3)	9 (9.8)	
NBW	1236 (84.5)	82 (89.1)	
HBW	60 (4.1)	1 (1.1)	
Mean LBW weight ± SD, g	2032 ± 400	2041 ± 415	0.953
Mean NBW weight ± SD, g	3225 ± 349	3187 ± 341	0.327
Mean HBW weight ± SD, g	4199 ± 193	4200	-
Delivery mode, n (%)			0.504
Vaginal	733 (50.1)	47 (51)	
Cesarean section	681 (46.6)	44 (48)	
Operative vaginal	48 (3.3)	1 (1)	
Parity, n (%)			0.163
0	636 (47.1)	40 (56.3)	
1	714 (52.9)	31 (43.7)	
Previous children, median (IQR)	0 (0–1)	1 (0–2)	0.18 **
AEFIs, n (%)	0 (0)	-	-

Abbreviations. GA, gestational age; LBW, low birth weight; NBW, normal birth weight; HBW, high birth weight; AEFIs, adverse events following immunization; parity 0 = primiparous, 1 = multiparous. The post-term group was not included in inferential comparisons because of the very small sample size. For maternal parity, data were available for 1350/1462 immunized neonates and 71/92 non-immunized neonates, and percentages were calculated using non-missing data as the denominator. For the number of previous children, data were available for 1341/1462 immunized neonates and 38/92 non-immunized neonates; for primiparous women the value was 0. * Fisher-Freeman-Halton exact test (birth weight category); ** Mann-Whitney U test (number of previous children).

## Data Availability

The original contributions presented in this study are included in the article. Further inquiries can be directed to the corresponding author.
